# Role of Kynurenine and Its Derivatives in the Neuroimmune System

**DOI:** 10.3390/ijms25137144

**Published:** 2024-06-28

**Authors:** Makoto Fujikawa, Masashi Ueda, Kenta Maruyama

**Affiliations:** Department of Pharmacology, School of Medicine, Aichi Medical University, Nagakute 480-1195, Aichi, Japan

**Keywords:** tryptophan, kynurenine pathway, neuroimmunology, sepsis, brain

## Abstract

In recent years, there has been a growing realization of intricate interactions between the nervous and immune systems, characterized by shared humoral factors and receptors. This interplay forms the basis of the neuroimmune system, the understanding of which will provide insights into the pathogenesis of neurological diseases, in which the involvement of the immune system has been overlooked. Kynurenine and its derivatives derived from tryptophan have long been implicated in the pathogenesis of various neurological diseases. Recent studies have revealed their close association not only with neurological disorders but also with sepsis-related deaths. This review provides an overview of the biochemistry of kynurenine and its derivatives, followed by a discussion of their role via the modulation of the neuroimmune system in various diseases.

## 1. Introduction

In the 19th century, the discovery of kynurenic acid (KYNA), along with the determination of its chemical structure and elucidation of its function, marked the beginning of the elucidation of the “kynurenine (KYN) pathway”, which holds great importance in neuroscience [1]. In 1931, Yashiro Kotake identified KYN in the urine of dogs injected with high amounts of tryptophan (Trp) [2]. In 1978, Lapin discovered that quinolinic acid (QUIN), a derivative of KYN, induced seizures in mice when administered into the brain ventricles [3]. KYN and its derivatives are important regulators of neuroimmune signaling via neurotransmitters and cytokines, forming an extensive network [4]. QUIN has recently been implicated as a direct cause of sepsis-related death [5]. This suggests that KYN and its derivatives play a pivotal role in the etiology of various diseases involving the neuroimmune system. This review outlines the role of KYN and its derivatives in the central nervous system (CNS) and related diseases. First, the metabolic regulation of KYN and its general actions are discussed, followed by the physiological functions of KYN, its pathological relevance in neurological diseases and sepsis, and the associated therapeutic challenges.

## 2. Overview of KYN Metabolism

Trp, the initial substrate of the KYN pathway, is an essential amino acid. Approximately 95% of Trp is utilized by the KYN pathway, whereas the remaining is metabolized for the synthesis of compounds, such as serotonin (5-HT), which plays a crucial role in neurotransmission [6]. Similar to 5-HT, metabolites of the KYN pathway have various functions and are known to act on the CNS, as mentioned previously. The first enzyme to metabolize Trp in the KYN pathway is either tryptophan 2,3-dioxygenase (TDO) or indoleamine 2,3-dioxygenase (IDO). IDO has two isoforms: IDO1 and IDO2. The deletion of both isoforms did not change the Trp level in the brain [7], but the deletion of TDO resulted in a ten-fold increase in the brain Trp level [8]. This indicates that TDO plays a significant role in Trp metabolism under normal conditions [9]. QUIN, the end-product of the KYN metabolic pathway, is a major substrate for de novo nicotinamide adenine dinucleotide (NAD^+^) synthesis. For instance, 60 mg of Trp resulted in the synthesis of 1 mg of niacin [10]. When the sympathetic nervous system is activated by stress, the brain Trp level increases and is influenced by catecholamine levels and blood–brain barrier (BBB) permeability [11]. Deficiency of Trp, an essential amino acid, impairs cognitive functions such as discrimination learning, episodic memory, cognitive flexibility, and reward learning abilities [12]. Trp depletion results in a surplus of free tryptophanyl transfer RNA (tRNA), which activates GCN2 kinase and triggers various cellular responses. Immune stimulation enhances Trp metabolism; consequently, Trp deficiency may inhibit the growth of infectious microorganisms and certain immune cells [13]. As mentioned above, the amount of Trp in the body affects an organism through various pathways, including the KYN metabolic pathway, which is responsible for 95% of Trp metabolism and is a major determinant of Trp level in the body.

## 3. Metabolites of the KYN Pathway

**Figure 1:**
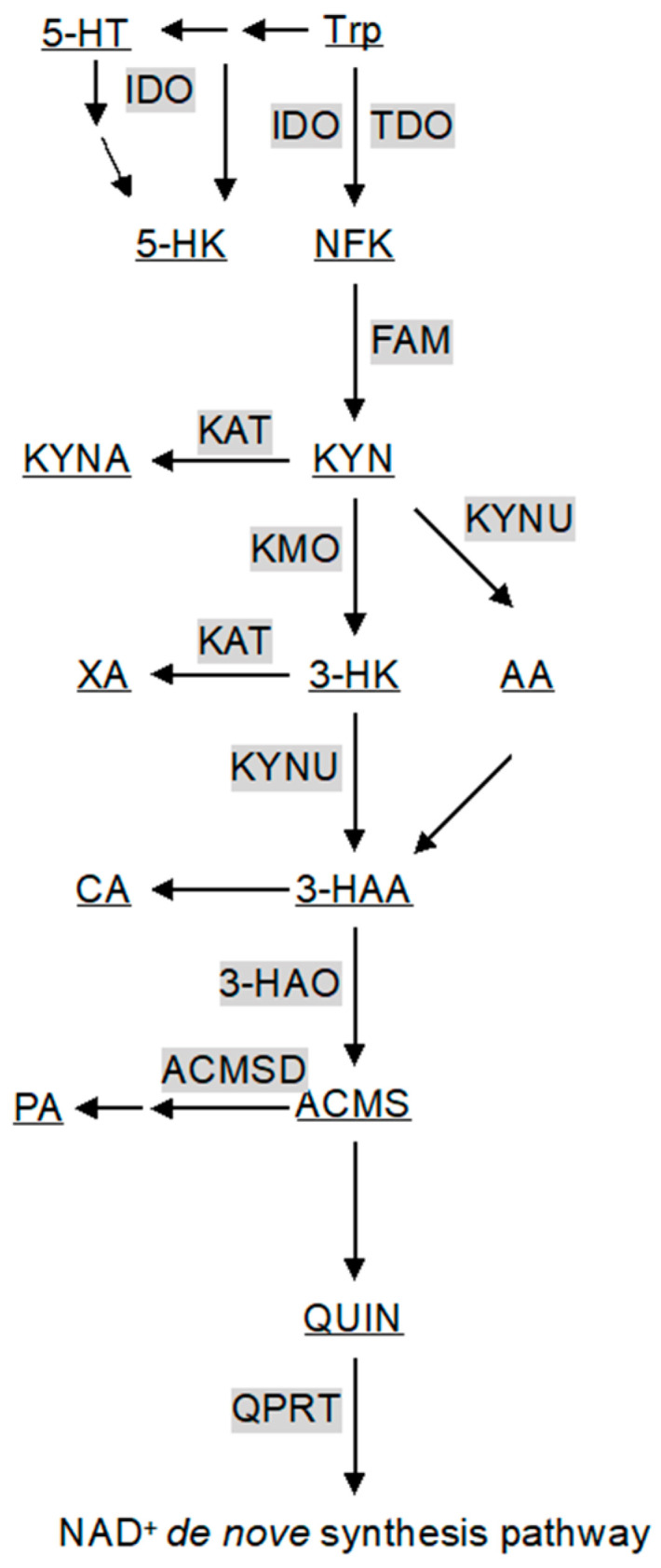
A schematic diagram of the kynurenine pathway. Gray boxes and underlining indicate enzymes catalyzing reactions (arrows) and metabolites of the kynurenine pathway. All abbreviations are shown in Table 1 and text.

Despite the significant effects of KYN derivatives on the CNS, 90% of the initial reactions of KYN metabolism occur in the liver, where TDO catalyzes the conversion of Trp to *N*′-formylkynurenine (NFK) [14]. Trp metabolism by TDO and IDO in the CNS is much lower than that in the periphery; 60% of all Trp metabolites present in the CNS are transported from the peripheral tissues, especially from the liver via the circulatory system [15]. In particular, 60–80% of KYN in the brain originates from outside the CNS [16]. Therefore, transport through the circulatory system and permeability through the BBB are critical for controlling KYN metabolite levels in the CNS. Among KYN metabolites, polar compounds such as kynurenic acid (KYNA), QUIN, and 3-hydroxyanthranilic acid (3-HAA) have low permeability through the BBB. In contrast, nonpolar Trp, KYN, and 3-hydroxykynurenine (3-HK) can enter the brain from glial cells via the large-neutral amino acid transporter (LAT) [16]. As Trp can only cross the BBB in its free form via serum albumin, the total amount of free Trp determines its uptake by the CNS [17]. Stress and exercise, both of which activate the sympathetic nervous system and increase free fatty acids (FFAs) in the blood, can affect Trp release [18]. FFAs bind antagonistically to serum albumin and Trp, resulting in more free Trp, which can then pass through the BBB into the CNS [19,20]. Other Trp metabolites also bind to plasma proteins, thereby limiting their translocation to the CNS [16]. Systemic inflammation promotes the translocation of KYN to the brain [21]. Thus, the synthesis and release of KYN derivatives in the liver, as well as their binding and dissociation from serum albumin and other substances, influence their BBB permeability and regulate KYN concentration in the CNS. KYN is converted to KYNA in astrocytes [22] and to QUIN in microglia and neurons [23], ensuring the presence of these metabolites in the CNS despite their low BBB permeability. However, under certain conditions, QUIN can cross the BBB or be transported by macrophages. Indeed, 50–70% of QUIN has been reported to cross the BBB in an experiment involving subcutaneous injection of labeled QUIN [24]. Therefore, it is often misunderstood that QUIN (and KYNA) cannot cross the BBB. Circulatory levels of blood Trp and KYN derivatives vary under different pathophysiological conditions. For example, the blood Trp level in pregnant women declines before and during parturition and rapidly recovers 2–4 days after delivery. Similarly, the KYN/Trp ratio is high during delivery and restores to baseline within a few days postpartum. However, in women who develop postpartum depression, the blood Trp level and the KYN/Trp ratio are not recovered and differ significantly from women who do not develop depression [25,26]. A recent study analyzing urinary metabolites in patients with coronavirus disease 2019 (COVID-19) found markedly elevated concentrations of KYN, 3-HK, and 3-HAA, with KYN levels correlating with COVID-19 severity [27]. Furthermore, the blood KYN/Trp ratio was significantly elevated in patients with COVID-19, and IDO2 expression was induced in peripheral blood mononuclear cells (PBMCs) and in the postmortem brains of patients with post-acute sequelae of SARS-CoV-2 infection (PASC), suggesting a link between PASC and KYN metabolism [28]. Thus, blood levels of KYN derivatives and the Trp/KYN ratio are important biomarkers of psychiatric and infectious diseases. As blood levels of these derivatives significantly affect their CNS levels, there is potential for therapeutic interventions to control these levels using drugs that do not cross the BBB (Table 1, Figure 1). In addition to the major metabolites, others that are minorly associated with the kynurenine pathway will be mentioned. Cinnabarinic acid (CA) is produced via the non-enzymatical catabolism of 3-HAA. As of June 2024, there have been 62 reports focused on CA in PubMed, so the physiological functions remain defined. From a small number of reports, CA appears to act as a neuroprotective factor because it can be an agonist for metabotropic glutamate (mGlu) receptor 4 and can be anti-exitotoxic [29]. Xanthurenic acid (XA), which acts as an mGlu receptor similar to CA, is produced mainly by KAT II from 3-HK. While this suggests that XA is related to schizophrenia, administration of XA by peritoneal or intracerebroventricular injection promotes dopamine release in the brain; therefore, it seems that XA is neuroprotective [30,31]. Moreover, it is indicated that XA can protect against iron deposition in the amyloid plaques of Alzheimer’s disease because XA forms a complex by coordinating with iron or zinc ions [32,33]. Picolinic acid (PA) is produced by aminocarboxymuconate-semialdehyde-decarboxylase (ACMSD) catalyzing 3-HAA, like CA. It is reported that PA, similar to XA, can bind to iron and zinc and can be a neuroprotective agent [34]. PA administration alleviates the lethality of mice infected with *Candida albican* [35]. PA stimulates macrophages and promotes MIP-1α and MIP-1β release [36]. Additionally, it is known that PA differentiates mesenchymal stem cells to bones in vivo; thus, it is expected to be applied as a therapeutic agent [37]. Anthranilic acid (AA) is produced by kynureninase from KYN and is oxidized to 3-HAA, resulting in joining the kynurenine pathway. AA, with 3-HAA, decreases IDO activity and lowers KYN production, leading to the downregulation of AhR stimulation. AhR signaling is associated with the suppression of vasculitis, so AA is able to prevent arteriosclerosis [38] (Figure 1).

## 4. Enzymes of the KYN Pathway

Tryptophan 2,3-dioxygenase and indoleamine 2,3-dioxygenase. The rate-limiting step in the KYN metabolic pathway is the initial reaction converting Trp to NFK, catalyzed by tryptophan 2,3-dioxygenase (TDO) and indoleamine 2,3-dioxygenase (IDO) [39,40] (Figure 1). Stress-induced secretion of glucocorticoids from the adrenal cortex enhances TDO transcription [39,40]. Under stressful conditions, increased Trp consumption by TDO reduces Trp availability for 5-HT synthesis, leading to decreased 5-HT production [9]. However, a persistent increase in glucocorticoid levels can desensitize glucocorticoid receptors and suppress TDO expression in the liver [41]. Human and mouse TDOs exist as heme iron-free apoenzyme and heme-containing holoenzyme in a 1:1 ratio, indicating that heme content regulates TDO activity [42,43]. In patients with porphyria, inadequate heme pools decrease TDO activity and increase blood Trp and brain 5-HT [44]. TDO is allosterically inhibited by NAD(P)H produced via the KYN pathway in the de novo NAD^+^ synthesis pathway [45]. Additionally, intermediate metabolites of the KYN pathway, such as KYNA and 3-HAA, inhibit TDO activity [46]. However, this inhibitory effect was not observed in rats, suggesting species-specific differences that require further investigation [47]. Unlike TDO, IDO is not induced by glucocorticoids [48,49]. IDO is subjected to substrate inhibition at high Trp concentrations and has lower substrate specificity than TDO, allowing it to metabolize 5-HT intermediates such as 5-hydroxytryptophan to 5-hydroxykynurenine (5-HK) [50,51,52]. This pathway diversion depletes 5-HT synthesis substrates. IDO is transcriptionally and translationally upregulated by interferon-gamma (IFN-γ) [53,54]. IFN-γ-induced IDO transcription is inhibited by interleukin-4 (IL-4), IL-10, and transforming growth factor-beta (TGF-β) and enhanced by IL-1β and tumor necrosis factor-alpha (TNF-α) [55]. Lipopolysaccharide (LPS) induces IDO1 expression via inflammatory cytokines through Toll-like receptor 4 (TLR4) signaling [56]. IDO is reversibly inhibited by nitric oxide (NO) through the formation of a heme iron–NO–Trp complex within the IDO protein [57,58]. The brain levels of Trp and KYN derivatives in IDO2-knockout mice are the same as those in wild-type mice, suggesting redundancy or compensation by other pathways [8]. Although vertebrates possess both IDO1 and IDO2, the human IDO2 ortholog is less active, in contrast to some fish species with high IDO2 activity [59]. However, the physiological significance of IDO2 in humans remains unclear. Thus, although TDO and IDO catalyze the same initial step in KYN metabolism, their different expression patterns and regulatory mechanisms result in distinct pathophysiological roles.

Kynurenine aminotransferase. Kynurenine aminotransferase (KAT) converts KYN to KYNA (Figure 1). Humans possess four KAT isoforms (I-IV); however, KAT II is the primary enzyme that generates KYNA in the brain. KAT II exhibits low substrate specificity and can catalyze the transamination of multiple α-keto acids, such as pyruvate, oxaloacetate, and α-ketoglutarate, in addition to KYN [60]. KAT II is also known to play a major role in the synthesis of xanthurenic acid (XA), an iron chelator that binds heme and iron from 3-HK in the brain [61,62]. KYNA has a limited ability to cross the BBB, making the regulation of KAT II activity critical for maintaining KYNA levels in the brain. Exercise activates the PGC-1α and PPARα/δ pathways in skeletal muscles, increasing KAT expression and promoting KYNA production, which has neuroprotective effects [63]. Consequently, the KYN/KYNA blood ratio is reduced, thus protecting the brain from stress-induced injuries related to depression. Under normal conditions, the synthesis reaction of KYNA with KYN as a substrate is much less efficient than that of 3-HK with KYN as a substrate because the catalytic activity of kynurenine 3-monooxygenase (KMO) for 3-HK is two orders higher than that of kyunureninase (KYNU) for KYNA. Energy metabolic intermediates, such as pyruvate, oxaloacetate, and α-ketoglutarate, promote KYNA synthesis due to their α-keto acid structure, and KAT catalyzes the amino group transfer to them. Under conditions such as glucose deprivation, the levels of these intermediates are reduced, leading to decreased KYNA synthesis [64].

Kynurenine 3-monooxygenase. KMO is a flavoprotein that requires vitamin B2 (riboflavin) for the catalysis of the conversion of KYN to 3-HK (Figure 1). Vitamin B2 deficiency decreases KMO activity, reduces 3-HK levels, and increases KYN and KYNA levels in the urine [65]. A girl with a presumed genetic defect in KMO exhibited pellagra-like symptoms due to niacin deficiency, with urinary KYN and KYNA levels 10–1000 times higher than normal and lower urinary 3-HK levels [66]. She also exhibited behavioral symptoms, such as tearfulness, withdrawal, anxiety, and depression, suggesting that KMO deficiency may cause deviations in KYN derivatives, including niacin (and NAD^+^), contributing to these symptoms. Similarly, KMO-knockout mice exhibited increased blood levels of KYN and KYNA, decreased 3-HK levels, and decreased blood and hepatic QUIN levels while maintaining brain QUIN levels, likely due to bypassing KMO via KYN-anthranilic acid (AA)-3-HAA [67]. The inflammatory cytokine IL-1β upregulates KMO transcription in the human hippocampus [68], and LPS stimulation increases KMO activity, indicating a shift in the KYN metabolic pathway from KYNA toward QUIN synthesis [69]. Thus, inflammatory stimuli promoted QUIN synthesis from Trp.

Kynureninase. KYNU is widely expressed in tissues and requires vitamin B6 (VB6) for its activity. VB6 deficiency causes niacin deficiency and pellagra symptoms, similar to the effects of riboflavin deficiency on KMO [70,71]. Several splicing variants of KYNU exist, catalyzing both the conversion of 3-HK to 3-HAA and KYN to AA; the Km for KYNU is 77 μM for 3-HK and 1000 μM for KYN, making the conversion of KYN to AA less efficient than that of 3-HK to 3-HAA [72] (Figure 1). As KMO’s Km for KYN is approximately 20 μM, KYN metabolism typically does not directly lead to AA or KYNA synthesis but rather to QUIN via 3-HK. Additionally, AA produced from KYN by KYNU can be non-enzymatically hydroxylated to 3-HAA, allowing QUIN synthesis via AA even if KMO is dysfunctional.

3-Hydroxyanthranilate 3,4-dioxygenase and others. 3-Hydroxyanthranilate 3,4-dioxygenase (3-HAO) catalyzes the conversion of 3-HAA to 2-amino-3-carboxymuconic acid-6-semialdehyde (ACMS) (Figure 1). This reaction is the key step in the KYN pathway. Subsequently, ACMS undergoes a non-enzymatic reaction to form QUIN. 3-HAO contains non-heme iron as a cofactor, which is essential for its activity. It is activated by Fe^2+^ and inhibited by iron chelation, indicating that iron concentration can influence the enzyme’s activity [73]. This suggests that fluctuations in iron levels can affect QUIN concentration in the brain, potentially affecting neurodegenerative and psychiatric diseases. QUIN is further converted to nicotinic acid mononucleotide (NaMN), integrating the KYN pathway into the de novo NAD^+^ synthesis pathway. NAD^+^ plays a crucial role in various cellular processes, including ATP synthesis, regulation of gene expression through sirtuins, and DNA repair via poly ADP-ribose polymerases (PARPs). Increased IDO expression, stimulated by IFN-γ in macrophages, can activate PARPs to repair DNA damage caused by free radicals generated during enhanced energy metabolism [74]. The rate-limiting enzyme of the KYN pathway, TDO, is inhibited by NAD(P)H produced in the de novo NAD^+^ synthesis pathway [45]. In contrast, IDO does not undergo catabolic inhibition during inflammation. In studies involving TDO-knockout (TDO-KO) mice, different responses have been observed depending on dietary niacin availability. When these mice were fed a diet containing niacin, they maintained a normal body weight and liver NAD^+^ levels. However, in the absence of niacin, the urinary levels of KYN, KYNA, and 3-HK were significantly elevated compared with those in wild-type mice. The absence of niacin also resulted in lower total amounts of QUIN and niacin derivatives in the urine. These findings indicate that while extrahepatic IDO and subsequent enzymes can process KYN to 3-HK, they are not sufficient for producing QUIN and completing de novo NAD^+^ synthesis in extrahepatic tissues [75].

## 5. KYN Metabolites as Receptor Ligands

KYN metabolites act as agonists or antagonists of various receptors, including those in neurons. For instance, KYN acts as an agonist for the human aryl hydrocarbon receptor (AhR), which plays a role in cytokine (IL-1β and IL-6) secretion [76]. However, the ligand selectivity of AhR differs significantly between humans and mice, emphasizing the need for cautious interpretation [77,78]. KYNA is a potent inhibitor of *N*-methyl-D-aspartate receptors (NMDARs), blocking glutamate- and glycine-binding sites and exhibiting neuroprotective effects [79,80]. Additionally, KYNA negatively affects the nicotinic acetylcholine receptor α7 (α7nAChR) [81,82]. By binding to G-protein-coupled receptor 35 (GPR35), KYNA inhibits inflammatory pathways by blocking N-type Ca^2+^ channels in sympathetic neurons and astrocytes [83,84,85]. Furthermore, KYNA binds to GPR35 and translocates it to the mitochondria, inhibiting the reverse reaction of ATP synthase and contributing to hypoxia tolerance [86]. The effects of KYNA on multiple receptors suggest that it has diverse functions based on excitatory or inhibitory synaptic transmission patterns. In contrast, QUIN acts as an NMDAR agonist and leads to excitotoxicity [87]. Both KYN and KYNA act as ligands for AhR, which regulates KYN metabolism. The regulation of IDO2 expression by AhR is linked to the immune response to self-antigens [88]. Disruption of the AhR gene in mice increases KAT II expression in the cortex and striatum, resulting in elevated KYNA levels and protection against excitotoxicity and oxidative stress [89]. In the brain–gut axis, Trp metabolism by the gut microbiota influences KYN levels as well as the serum KYN/Trp ratio. A systematic review suggested that probiotics and prebiotics could affect KYN levels and the ratio [90]. Additionally, enterobacterial tryptophanases catalyze the conversion of food-derived Trp to indole derivatives, which can activate or inactivate AhR and impact the KYN metabolic pathway, thereby affecting enzyme expression, blood KYN levels, and the KYN/Trp ratio, ultimately influencing KYN homeostasis in the CNS.

## 6. KYN Metabolites in the Pathology of Various Neurodegenerative Disorders

Huntington’s Disease. An imbalance in KYN metabolism can lead to neurotoxicity. Huntington’s disease (HD), caused by the expansion of CAG repeats in exon 1 of the huntingtin (Htt) gene, is a pertinent example [91]. Administration of QUIN to the striatum exacerbated neuronal cell death via excitotoxicity in an HD mouse model, implicating NMDAR-mediated excitotoxicity in HD pathology [92]. Studies have long implicated elevated levels of 3-HK and QUIN in the brains of patients with HD, particularly during the early stage of the disease [93,94]. KYN is metabolized to KYNA by KAT, and the KYN/KYNA ratio increases in the putamen of patients with HD. Enzyme activity assays have indicated significantly decreased KAT I and KAT II activities in the putamen of patients with HD compared with controls [95,96]. These findings suggest that an imbalance in KYN metabolism, including increased levels of 3-HK and QUIN, stems from abnormalities in the KYN pathway. Similarly, increased levels of 3-HK and QUIN were observed in the striatum and cerebral cortex of HD model mice [97]. Following [^3^H] KYN administration to the striatum of R6/2 mice (a well-known HD model), [^3^H] 3-HK levels were elevated in R6/2 mice compared with those in wild-type mice. Enzyme activity analysis revealed increased KMO activity and decreased KYNU activity in the brain tissues, such as the cerebral cortex of these mouse models [98]. In vitro analysis using primary cultured neurons showed that 3-HK induces neuronal cell death in the striatum and cerebral cortex but not in the cerebellum, indicating the region-specific toxicity of 3-HK. The o-aminophenol structure of 3-HK produces reactive oxygen species (ROS) through auto-oxidation, and antioxidants inhibit 3-HK toxicity in neurons [99]. Inhibition of histone deacetylase reduces 3-HK levels and KMO activity in primary cultured microglial cells of R6/2 mice [100]. This suggests that mutant Htt causes transcriptional abnormalities mediated by histone modifications in microglia, leading to increased 3-HK levels. These mouse models and in vitro analyses indicated that (1) abnormalities in the activities and expression levels of KYN metabolic enzymes lead to increased 3-HK and QUIN levels, and (2) these increased KYN metabolites induce neuronal toxicity through NMDAR-mediated excitotoxicity and ROS-related effects. In summary, although the precise molecular mechanism remains unclear, the expansion of CAG repeats in the Htt gene enhances KMO activity in the brain, resulting in excessive 3-HK and QUIN production. 3-HK enters neuronal cells in a region-specific manner, such as in the striatum, leading to the overproduction of ROS. Increased QUIN expression is associated with NMDAR in neurons, inducing excitotoxicity. A decreased KYNA level due to abnormal KAT activity reduces neuroprotective effects by diminishing NMDAR inhibition, leading to neuronal cell death. Understanding the role of KYN metabolites in the molecular pathology of HD is crucial for elucidating the relationship between Trp metabolic abnormalities and neurodegenerative disorders (Table 2).

Alzheimer’s Disease. Alzheimer’s disease (AD) accounts for the largest proportion of dementia cases, characterized by the formation of amyloid-β (Aβ) aggregates and tau protein phosphorylation, which are central to its pathology. In postmortem brains of patients with AD, increased QUIN immunoreactivity is observed in the hippocampal dentate gyrus, with Aβ aggregates showing positivity for both QUIN and IDO [101]. Additionally, QUIN co-localizes with tau protein near neuritic plaques, and its administration to human primary cultured neurons promotes tau phosphorylation and aggregation [113]. These findings suggest that QUIN, localized within Aβ aggregates, contributes to neurotoxicity via tau protein phosphorylation. Oral administration of a KMO inhibitor to AD model mice increased KYNA levels in the brain and improved AD-like behavioral phenotypes [114], indicating the involvement of KYN metabolic abnormalities in AD pathology. Notably, increased QUIN immunoreactivity is observed not only in neurons but also in microglia and astrocytes in the hippocampus [101], suggesting that microglia-produced QUIN contributes to neuronal cytotoxicity in AD. It is also known that the proportion of QUIN-positive mononuclear cells and 3-HK levels in the plasma are elevated in the peripheral tissues of patients with AD [102,103]. When the permeability of the BBB increases due to systemic inflammation, the peripheral KYN level increases, subsequently increasing brain QUIN levels [21]. These results suggest that QUIN from the peripheral tissues migrates to the CNS and contributes to neuronal degeneration in AD. In summary, increased QUIN levels, whether derived from peripheral tissues or produced within the CNS, correlate with Aβ aggregate formation and tau protein phosphorylation, leading to neuronal degeneration and death in AD. Understanding the role of KYN metabolites in AD is crucial to elucidate the link between Trp metabolic abnormalities and neurodegenerative disorders (Table 2).

Parkinson’s Disease. Abnormalities in KYN metabolites are implicated in neurodegeneration observed in Parkinson’s disease (PD). Increased 3-HK levels and decreased KYN and KYNA levels have been reported in patients with PD [104]. Recent genetic analyses have identified a correlation between missense mutations in KMO and late-onset PD [115]. Decreased expression of KAT I in the pars compacta of the substantia nigra and reduced KAT II activity in the cerebral cortex were observed in PD model animals [105]. These findings suggest that KAT dysfunction contributes to the pathology of PD by decreasing KYNA levels, which, in turn, reduces neuroprotection. Given that KMO inhibition reduces neurotoxicity, BBB-permeable KMO inhibitors have shown therapeutic potential [116]. Thus, decreased KYNA levels in the brain, primarily caused by KAT dysfunction, are believed to play a crucial role in the pathology of PD by diminishing the neuroprotective effects (Table 2).

Multiple Sclerosis. Multiple sclerosis (MS) is a chronic inflammatory demyelinating disease of the CNS, and its onset is linked to Epstein–Barr virus infection [117]. In MS, activated T cells extensively infiltrate the CNS, leading to demyelination. IDO, an enzyme that metabolizes Trp to KYN in the brain, suppresses T-cell activation. This suppression aligns with the finding that the inhibition of IDO exacerbates immune responses and worsens MS symptoms in animal models [106,107]. Notably, the administration of a synthetic derivative of AA, a Trp metabolite, ameliorated autoimmune responses in MS model mice [118]. Additionally, IFN is known to activate IDO [110], and IFN administration has shown therapeutic efficacy in MS. These findings underscore the importance of IDO activity in regulating T-cell function and its role in the pathology of MS (Table 2).

## 7. Involvement of KYN Metabolites in Psychiatric Disorders

Selective serotonin reuptake inhibitors (SSRIs) and monoamine oxidase (MAO) inhibitors are widely used for the treatment of psychiatric disorders such as depression and schizophrenia. Historically, 5-HT has been the primary focus for understanding the pathology of these disorders and serves as the main target for these drugs. However, recent research has highlighted the significant role of KYN metabolites in the molecular pathology of psychiatric disorders [119]. Emerging evidence suggests that an imbalance in Trp metabolism, which leads to altered levels of KYN metabolites, contributes to the pathophysiology of these conditions. Understanding the involvement of the KYN pathways offers new insights and potential therapeutic targets beyond traditional 5-HT-centric approaches.

Depression. Depression is a mental illness that is often triggered by excessive stress and neuroinflammation [120]. Its pathology is primarily linked to functional abnormalities in monoaminergic systems, particularly 5-HT, in the CNS. Current therapeutic agents, such as SSRIs and MAO inhibitors, function by increasing 5-HT levels. Although Trp administration can alleviate the symptoms of depression and anxiety through its metabolites 5-HT and KYN, approximately 30% of patients with major depressive disorder (MDD) are resistant to SSRIs. This resistance highlights the need to develop novel therapeutic agents targeting different molecular pathways. The link between depression and inflammation is well documented. For instance, IFN therapy for chronic hepatitis C can induce depression. In patients with IFN-induced depression, single-nucleotide polymorphisms (SNPs) in genes encoding KYN metabolism enzymes, such as formamidase and KYNU, have been identified [121]. Additionally, an elevated plasma KYN/Trp ratio is correlated with the severity of depression and anxiety in patients with depression and IFN-induced depression [25,108], suggesting the involvement of KYN metabolic abnormalities in depression pathology. Postmortem studies of patients with depression have revealed increased microglial activity and a higher number of QUIN-positive cells in the anterior cingulate cortex, along with a decrease in the number of astrocytes in the hippocampus [109,122]. These findings are mirrored in animal models of depression [122,123]. Moreover, an increased expression level and activity of IDO has been observed in the lungs and brain of a mouse model of depression induced by bacterial infection. Notably, these depression-like phenotypes and the elevated IDO expression were mitigated in IFN-γ receptor-deficient mice [110]. These studies suggest that the cytokine-mediated activation of IDO in the peripheral tissues contributes to depressive symptoms. The diverse findings on KYN metabolic abnormalities in depression suggest a possible mechanism by which the peripheral IFN activates IDO in the CNS, leading to increased QUIN levels in the brain and the subsequent activation of NMDA receptors. Understanding these pathways will offer new avenues for therapeutic interventions targeting KYN metabolites in depression (Table 2).

Schizophrenia. Schizophrenia is a complex psychiatric disorder characterized by positive (hallucinations and delusions) and negative (social withdrawal and anhedonia) symptoms. Treatment commonly involves dopamine receptor blockers for positive symptoms and 5-HT_2A_ blockers for negative symptoms. The two major hypotheses for the etiology of schizophrenia are the glutamate hypothesis, which involves NMDAR dysfunction, and the dopamine hypothesis [124]. KYNA, a metabolite of the KYN pathway, functions as an antagonist of endogenous NMDAR in the human CNS, suggesting its potential involvement in the molecular pathology of schizophrenia. Supporting this, significantly elevated KYNA levels have been observed in the prefrontal cortex of patients with schizophrenia [111]. Furthermore, a decrease in KMO activity in this brain region is thought to contribute to the elevated KYNA levels, implicating abnormalities in the KYN metabolic pathway. These findings are consistent with increased KYNA concentrations in the prefrontal cortex of animal models of cognitive dysfunction [112]. The glutamate hypothesis of schizophrenia suggests that reduced NMDAR function leads to disorder symptoms. Given that KYNA is an NMDAR antagonist, increased levels in patients with schizophrenia may contribute to the observed NMDAR hypofunction. This suggests that therapeutic strategies aimed at modulating KYNA levels or its pathways could potentially alleviate some of the symptoms of schizophrenia. Further research on the precise role of KYNA and other KYN metabolites in schizophrenia is essential to develop novel and effective treatment options for this debilitating disorder (Table 2).

## 8. Involvement of KYN Metabolites in Septic Shock

**Figure 2:**
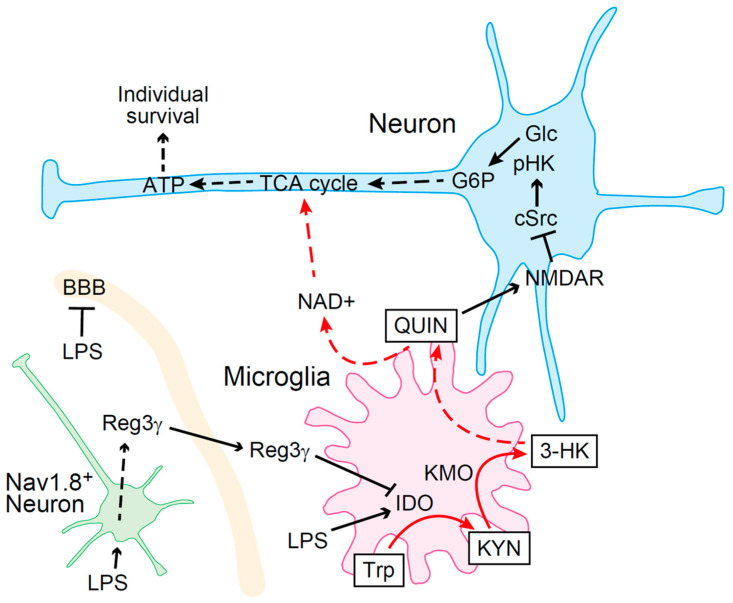
Schematic model of nociceptor-derived Reg3γ-mediated tolerance to LPS. Systemic inflammation caused by LPS increases QUIN levels by upregulating IDO expression in microglia, which induces metabolic arrest in neurons, thereby leading to death. Reg3γ is produced when nociceptors are stimulated by LPS. Nociceptor-derived Reg3γ crosses the BBB and targets microglia to suppress IDO expression, thereby preventing death (LPS, lipopolysaccharide; 3-HK, 3-hydroxykynurenine; ATP, adenosine triphosphate; BBB, blood–brain barrier; G6P, glucose-6-phosphate; Glc, glucose; KYN, kynurenine; LPS, lipopolysaccharide; NAD, nicotinamide adenine dinucleotide; NMDAR, *N*-methyl-D-aspartate receptors; pHK, phosphorylated hexokinase; QUIN, quinolinic acid; Trp, tryptophan; TCA, tricarboxylic acid).

Sepsis is a fatal condition that results from multiple organ failure because of dysregulation of host immune responses to pathogens and is one of the leading causes of death in intensive care units [125]. Despite the administration of anti-TNF-α antibodies and glucocorticoids to patients with sepsis, the mortality rate remains as high as 30% [126]. These findings suggest that the suppression of inflammation alone is insufficient to prevent death from sepsis. Recent studies have reported that KYN metabolites play a role in the maintenance of brain function and survival. The nociceptive system regulates immunity. In several mouse models of sepsis, genetic deletion or pharmacological inhibition of transient receptor potential vanilloid (TRPV) resulted in increased levels of inflammatory cytokines and vulnerability to hypotension [127,128,129]. As TRPV1 functions in immune cells, such as macrophages [128], we investigated whether the nociceptive system plays an important role in sepsis [5] using Nav1.8-Cre Rosa26DTA nociceptor-deficient mice [130]. The administration of LPS to Nav1.8-Cre Rosa26DTA mice induced death earlier than in controls. However, no significant differences were observed in plasma cytokine levels compared with those in wild-type mice. These results suggest that the cause of death was other than cytokine overproduction. Metabolomic analysis revealed that the KYN pathway was activated in Nav1.8-Cre Rosa26DTA mice following LPS administration. The QUIN level was significantly increased in the brain, and pharmacological IDO1 inhibition improved the survival rate of mice. Moreover, increased QUIN expression inhibited HK1 phosphorylation and glycolysis in neurons. HK1 phosphorylation by c-Src is essential for the activation of the glycolytic pathway [131]. We showed that LPS induces c-Src activation in neuronal cells as well as in immune cells [131], and QUIN inhibits LPS-mediated c-Src activation. Furthermore, nociceptors produce an antibacterial peptide, Reg3γ, upon LPS stimulation. After in vivo LPS administration, nociceptor-derived Reg3γ crosses the BBB and inhibits the expression of IDO1 in microglia. Mice lacking nociceptor-derived Reg3γ exhibit significantly higher mortality rates when exposed to LPS. This increased mortality is linked to the reduced phosphorylation of HK1 and diminished ATP production in the brain despite the presence of normal peripheral inflammatory responses. This metabolic failure is confined to the brain, where excessive QUIN production leads to the suppression of HK1 activity. Remarkably, central administration of Reg3γ enhances brain ATP production and significantly improves the survival rates of these mice. Thus, our findings identify nociceptor-derived Reg3γ as a critical microglia-targeted hormone, offering new insights into the mechanisms of resilience against septic death [5] (Figure 2).

## 9. Effects of Peripheral KYN Metabolites Produced by Inflammation on the Brain

Previous studies reported that QUIN and KYNA do not cross the BBB [16], while KYN can readily do so [21]. Notably, the exercise-induced activation of kynurenine aminotransferases (KATs) in skeletal muscle has been shown to reduce the amount of KYN transferred from the periphery to the brain, thereby alleviating depression [63]. This underscores the significant impact of peripheral KYN levels on brain function. It has also been reported that systemic KYN accounts for more than half of the KYN pool in the brain, and when systemic inflammation is induced by LPS, nearly all of the KYN pool in the brain is supplied from circulating blood-derived KYN [21]. Systemic inflammation, including sepsis, is also known to increase the permeability of the BBB and the activity of the KYN pathway in peripheral tissues, leading to the influx of KYN metabolites, which typically do not cross the BBB, into the brain [132,133]. The prevalence of depression in patients with an autoimmune disease such as rheumatoid arthritis (RA) is significantly higher than in the general population [134]. This increased incidence of depression in RA patients, along with the enhanced disruption of the BBB observed in animal models of RA, suggests a link between BBB disruption and the migration of KYN metabolites into the brain [135]. Conversely, it has been reported that exogenous KYNA in sepsis models provides neuroprotection by inhibiting BBB permeability through the suppression of neutrophil extracellular trap (NET) formation, indicating that some KYN metabolites may help prevent BBB disruption [136]. Despite these findings, much remains unknown about the importance of peripherally produced KYN metabolites on the brain. As mentioned above, early mortality in Nav1.8-Cre Rosa26DTA nociceptor-deficient mice during LPS-induced shock has been attributed to increased brain QUIN levels, and mortality in these mice is ameliorated by the central administration of an IDO1 inhibitor, suggesting that the peripheral influx of QUIN and other KYN metabolites into the brain during sepsis has a negligible effect on survival [5] (Figure 2). KYN in the brain is known to exacerbate depression and stroke pathophysiology by activating the AhR [137,138]. However, some reports suggest that AhR activation can prevent septic death and that KYN plays a protective role in ischemia, indicating the need for the careful interpretation of functions of KYN metabolites in the brain [139,140]. Understanding the mechanisms underlying pathological phenomena induced by changes in BBB permeability and the resultant alterations in brain concentrations of KYN metabolites caused by systemic inflammation and autoimmune diseases will be increasingly important.

## 10. Conclusions

This review highlights the role of KYN metabolites in the CNS and neuroimmune system, emphasizing their involvement in neurological diseases and sepsis. This underscores the complexity of KYN metabolic regulation in the CNS, as this review did not address the relationship between KYN metabolic control and diseases of the peripheral tissues or other physiological changes [119]. Further research is required to elucidate these complexities. Investigating the interplay between the neuroimmune system and KYN metabolites will provide novel insights into the mechanisms driving the development of these diseases. KYN and its metabolites are not gene products, necessitating indirect methods for analysis, such as studies on genetically deficient enzymes related to the KYN pathway. Additionally, quantitative changes in one KYN metabolite can influence the levels of other metabolites, highlighting the need for advanced research equipment that can detect low-molecular-weight compounds in the cells and tissues of diseased patients or animal models [141]. In humans, the quantitative control of metabolites can be relatively straightforward using similar compounds or enzyme inhibitors that have practical applications in clinical settings. Understanding the relationship between the neuroimmune system and KYN metabolism will facilitate the development of novel and effective therapeutic drugs for neurological diseases and sepsis [4].

## Figures and Tables

**Table 1:** Metabolites, enzymes, and regulators in the kynurenine pathway. The substrates and products of the enzymes in each reaction of the kynurenine pathway, as well as their regulators, are summarized.

Reaction	Substrate	Enzyme	Product	Regulator (Positive)	Regulator (Negative)
1	Trp	TDO	NFK	Trp, glucocorticoid, heme	3-HK, 3-HAA, NAD(P)H
1′	Trp/5-HT	IDO	NFK/5-HK	INF-γ, LPS, IL-1β, TNF-α	Trp, NO, IL-4, TGF-β
2	NFK	FAM	KYN	-	-
3	KYN	KMO	3-HK	IL-1β, LPS, VB2	-
3′	KYN/3-HK	KAT	KYNA/XA	A-keto acids *, PGC-1α/PRAPα/δ	KMO
4	3-HK/KYN	KYNU	3-HAA/AA	VB6	-
5	3-HAA	3-HAO	ACMS	non-heme iron	-
6	ACMS	non-enzymatic	QUIN	-	-
7 **	QUIN	QPRT	NaMN	--	

Abbreviations: Tryptophan 2,3-dioxygenase (TDO), indoleamine 2,3-dioxygenase (IDO), *N′*-formylkynurenine formamidase (FAM), kynurenine 3-monooxygenase (KMO), kynurenine aminotransferase (KAT), kynureninase (KYNU), 3-hydroxyanthranilate 3,4-dioxygenase (3-HAO), quinolinate phosphoribosyltransferase (QPRT), tryptophan (Trp), *N′*-formylkynurenine (NFK), 5-hydroxytryptamine (5-HT), kynurenin (KYN), 3-hydroxynurenine (3-HK), 5-hydroxykynurenine (5-HK), kynurenic acid (KYNA), xanthurenic acis (XA), 3-hydroxyanthranilic acid (3-HAA), anthranilic acid (AA), 2-amino-3-carboxymuconic acid-6-semialdehyde (ACMS), quinolinic acid (QUIN), nicotinic acid mononucleotide (NaMN), lipopolysaccharide (LPS), nitrogen oxide (NO), vitamin B2 (VB2), vitamin B6 (B6), * pyruvate, oxaloacetate, and α-keto-glutarate are classified as α-keto acids, ** the final product, QUIN, of kynurenine pathway goes through de novo NAD^+^ synthesis pathway.

**Table 2:** Alterations of kynurenine metabolites and kynurenine pathway-related enzymes in neurological and psychiatric disorders. The expression levels of kynurenine metabolites are altered in patients with several neurological and psychiatric disorders and their animal models. In patients and animal models of these disorders, kynurenine pathway-related enzymes show abnormal activities, and their expression levels are changed.

Disorder	Levels of Kynureine Metabolites	Activation and Expression Levels of Kynurenine Pathway-Related Enzymes
Huntington’s disease and its mouse model	Increased 3-HK, QUIN in the brain of patients [94] Increased KYN/KYNA ratio in the putamen of patients [95] Increased3-GK, QUIN in the brains of mice [97]	Decreased KAT I, KAT II activities in the putamen of patient [96] Increased KMO activity and decreased KYNU activity in the brains of mice [98]
Alzheimer’s diseases	Increased QUIN in the hippocampus of patients [101] Increased QUIN in the peripheral mononuclear cells of patients [102] Increased 3-HK in the plasma of patients [103]	Elevated IDO immunoreactivity in the hippocampus of patients [101]
Parkinson’s disease and its mouse model	Increased 3-HK in the brains of patients [104] Increased, KYN, KYNA in the brains of patients [104]	Decreased KAT I activity in the substantia nigra of mice [105] Decreased KAT II activity in the cerebral cortex of mice [105]
Multiple sclerosis mouse model	Changed KYN/TRP ration in mice [106]	Inhibition of IDO expression enhances multiple sclerosis symptoms in mice [107]
Depression and its mouse model	Increased KYN/TRP ratio in the plasma of patients [25,108] Increased QUIN in the anterior cingulate gyrus of patients [109]	Increased IDO expressions in the brains and lungs of mice [110]
Schizophrenia	Increased KYNA in the cortical brain regions of patients [111]	Decreased KMO activity in the cortical brain region of patients [112]

Abbreviations: 3-hydroxynurenine (3-HK), quinolinic acid (QUIN), kynurenin (KYN), kynurenic acid (KYNA), kynurenine aminotransferase (KAT), kynurenine 3-monooxygenase (KMO), kynureninase (KYNU), indoleamine 2,3-dioxygenase (IDO), tryptophan (TRP).

## Abbreviations

3-HAA, 3-hydroxyanthranilic acid; 3-HAO, 3-hydroxyanthranilate 3,4-dioxygenase; 3-HK, 3-hydroxykynurenine; α7nAChR, nicotinic acetylcholine receptor α7; Aβ, amyloid-β; AA, anthranilic acid; ACMS, 2-amino-3-carboxymuconic acid-6-semialdehyde; AD, Alzheimer’s disease; AhR, aryl hydrocarbon receptor; BBB, blood–brain barrier; CNS, central nervous system; COVID-19, coronavirus disease 2019; FFA, free fatty acid; GPR35, G protein-coupled receptor 35; HD, Huntington’s disease; IDO, indoleamine 2,3-dioxygenase; IFN-γ, interferon-γ; IL-4, interleukin-4; KAT, kynurenine aminotransferase; KMO, kynurenine 3-monooxygenase; KYNA, kynurenic acid; KYN, kynurenine; KYNU, kynureninase; LAT, large-neutral amino acid transporter; LPS, lipopolysaccharide; MAO, monoamine oxidase; MDD, major depressive disorder; MS, multiple sclerosis; NAD, nicotinamide adenine dinucleotide; NaMN, nicotinic acid mononucleotide; NMDARs, *N*-methyl-D-aspartate receptors; NO, nitric oxide; PARPs, poly ADP-ribose polymerases; PASC, post-acute sequelae of SARS-CoV-2 infection; PBMCs, peripheral blood mononuclear cells; PD, Parkinson’s disease; PNS, peripheral nervous system; QUIN, quinolinic acid; ROS, reactive oxygen species; SNP, single-nucleotide polymorphism; SSRIs, selective serotonin reuptake inhibitors; TDO, tryptophan 2,3-dioxygenase; TDO-KO, TDO-knockout; TGF-β, transforming growth factor-beta; TLR4, Toll-like receptor 4; TNF-α, tumor necrosis factor-alpha; tRNA, transfer RNA; Trp, tryptophan; TRPV, transient receptor potential vanilloid.
